# Spatiotemporal characteristics and driving forces of construction land expansion in Yangtze River economic belt, China

**DOI:** 10.1371/journal.pone.0227299

**Published:** 2020-01-24

**Authors:** Wenjie Cai, Tu Fangyuan

**Affiliations:** College of Public Administration, Huazhong University of Science and Technology, Wuhan, Hubei Province, PR China; Institute for Advanced Sustainability Studies, GERMANY

## Abstract

With rapid economic and population growth, construction land expansion in Yangtze River economic belt in China becomes substantial, carrying significant social and economic implications. This research uses Expansion Speed Index and Expansion Intensity Index to examine spatiotemporal characteristics of construction land expansion in the Yangtze River economic belt from 2000 to 2017. Based on a STIRPAT model, driving forces of construction land expansion are measured by Principal Component Analysis and Ordinary Least Square regression. The results show that: (1) there is a clear expansion pattern regarding the time sequence in provinces/cities of the Yangtze River economic belt, with rapid expansion in the initial stage, moderate expansion in the middle stage and rapid expansion in the later stage. (2) Spatial analysis demonstrates first expansion in the lower reaches in the early stage, rapid expansion of the upper reaches in the middle and later stage, and steady expansion of the middle reaches throughout the research period. (3)There are statistical significant correlations between construction land expansion and GDP, social fixed asset investments, population at the end of the year, population urbanization rate, per capita road area, and number of scientific and technological professionals as well as secondary and tertiary industry values. Of these factors, GDP, social fixed asset investments, population urbanization rate and second industry value are important common driving forces of construction land expansion in this region. The research findings have significant policy implications particularly on coordinated development of urban agglomerations and sustainable industry upgrading when construction land expansion is concerned.

## Introduction

With accelerated urbanization and rapid economic development in China, construction land expands at an increased speed and this trend will be sustained in the next few years [1, 2]. While construction land expansion provides space for urban construction and economic and social development, it also brings about a series of problems including over-occupation of rural arable land which threatens food security and destructions to ecological environment. Land expropriation in China has produced substantial number of landless farmers dissatisfied with resettlement and compensation arrangements, leading to several social problems [3]. It is of great significance to examine construction land expansion to promote economy, society and ecology development [4]. Spatial and temporal analysis of construction land expansion can reveal the expansion mechanisms, while quantitative methods can identify contributions of different driving forces of expansion, providing references for more targeted policies [5]. Characteristics and mechanisms of construction land expansion have attracted extensive attention from governments and scholars.

There is a wealth of research discussing construction land expansion in different countries. One seminal research was conducted in 1995 by the International Geosphere Biosphere Program and International Human Dimension Programme, which launched the Land Use/Cover Change research program and received enthusiastic responses from many international organizations and governments [6, 7]. Many scholars have carried out a large number of studies on the expansion of construction land, from perspectives of influences of changes in construction land [8], pattern measurement [9] and driving mechanism [10], and effectively exploring the influence of social and economic development [11], population size [12], transportation [13], living standard of residents [14] as well as urbanization process [15]. Kantakumar et al. [16] analyzed the spatiotemporal characteristics of construction land expansion in Pune, India, using remote sensing technology. The issue of urban expansion around Ulaanbaatar in Mongolia was examined by using feature-oriented spatial data [17]. Azhdari et al. [18] took Shiraz, Iran, as an example to discuss and analyze the relationship between spatial driving forces of urban expansion and socio-economic isolation. Garbarino et al. studied subalpine Larix decidua forests and analyzed driving factors of land use change [19]. Based on land-cover /use transitions in the binational Tijuana River watershed, Ojeda-Revah et al. discussed the spatial and temporal change pattern of land use [20].

Research on driving forces of land use change in China began in late 1990s. Most research focuses on the spatial characteristics and mechanism of construction land expansion, driving forces and measurement of expansion, as well as ecological environment responses to expansion [21, 22]. Research considers this issue nationwide [23, 24], at provincial level [25] and urban agglomeration [26], as well as in city area [27, 28] and marginal areas [29, 30]. Specifically for the Yangtze River economic belt, a giant economic belt straddling eastern and western China, more research focuses on construction land expansion in single cities or provinces. Hu et al. explored the driving factors of construction land expansion in Wuhan, Hubei province from 2003 to 2012 [31]. Taking large and medium-sized cities in Hubei province as an example, Yu [32] studied the influencing factors of construction land expansion in terms of time and space differences. Lv [33] discussed driving factors of urban construction land expansion in Hangzhou, Zhejiang province. Driving forces of urban construction land expansion in Wuxi city, Jiangsu province was studied by path coefficient analysis [34]. At present, there is inadequate research on construction land expansion and its driving force in the Yangtze River economic belt as a whole. This paper thus analyzes the expansion degree and explores main factors promoting construction land expansion in this region.

According to China statistical yearbook and China city statistical yearbook, Yangtze River economic belt's GDP reached 37.3 trillion Yuan, accounting for about 45.3% of China’s total economic output in 2017. With about 1/5 of the China’s land area, it contributed more than 2/5 of the country's total economic outputs [35, 36]. The outline of the development plan of the Yangtze River economic belt issued in September 2016 established a new development pattern of the Yangtze River economic belt with "one axis, two wings, three poles and multiple points". "One axis" means construction of a green development axis along the Yangtze River, relying on the golden waterway of the Yangtze River and the central role of Shanghai, Wuhan and Chongqing. "Two wings" respectively refer to the two major transportation channels of Hurui (Shanghai-Ruili, Yunnan) and Hurong (Shanghai-Chengdu, Sichuan), which are the basis for the development of the Yangtze River economic belt. "Three poles" refers to three urban agglomerations covering the Yangtze River delta, the middle reaches of the Yangtze River and Chengdu-Chongqing cities, giving full play of the radiation role of central cities. "Multi-point" means to take the supporting role of prefecture-level cities outside the three urban agglomerations, and to strengthen their economic interaction with central cities to drive regional economic development.

The Yangtze River economic belt has unique advantages and great development potential due to its advantageous geographical location and unique natural resources. Thus, revealing the spatiotemporal characteristics of construction land expansion in the Yangtze River economic belt and exploring its driving forces are of great significance to sustainable economic and social development of the Yangtze River economic belt and sustainable use of land resources.

Multiple methods have been used in analysis including gray relational degree analysis [37], STIRPAT model [38], Principal Component Analysis (PCA) [39], multiple regression analysis [40, 41] and co-integration analysis method based on Eviews software [4]. However, there is an issue of multicollinearity among variables in multiple regression analysis, while co-integration analysis cannot reveal whether there is a causal relationship between variables [5]. Additionally, it is unclear in Gray correlation results regarding the marginal contribution of driving factors. Tan and Ni [42] proposed that as it is difficult to quantify the driving forces influencing construction land expansion through a single correlation analysis and fitting result, this issue can be better examined through a particular perspective when establishing the analytical framework.

There are many types of analysis methods based on STIRPT model. Zhang et al. further indicated a combination of PCA and Ordinary Least Squares (OLS) regression to effectively eliminate the issue of multicollinearity among economic variables, and suggested to use this method in empirical research on driving forces of construction land expansion [5].This paper thus chooses to use this method. In terms of the research area, current research on construction land expansion and its driving force mainly focuses on single cities and provinces, less on regional city clusters, and particularly the Yangtze River economic belt. Further, by referring to relevant research and taking consideration of particular characteristics of the research region, this paper selects driving factors from the perspectives of urban economic development level, social policy intensity, population factors, urbanization level, urban road traffic conditions, technical level and industrial structure to analyze the correlation between them.

This paper builds a STIRPAT model, and combines PCA with OLS regression to investigate the expansion and driving forces of construction land expansion in provinces/cities of the Yangtze River economic belt in China, as well as the differences and coordinated development among provinces/cities in this region. The paper firstly introduced the research area and method. It then analyzed the spatiotemporal characteristics of construction land expansion in this region from 2000 to 2017 through measuring the Expansion Speed Index (ESI) and Expansion Intensity Index (EII) of construction land. The STIRPAT model was used to examine and compare the driving forces in each province/city. This was followed by conclusions from and discussions on research results.

## Data and method

### Area and data sources

**Fig 1:**
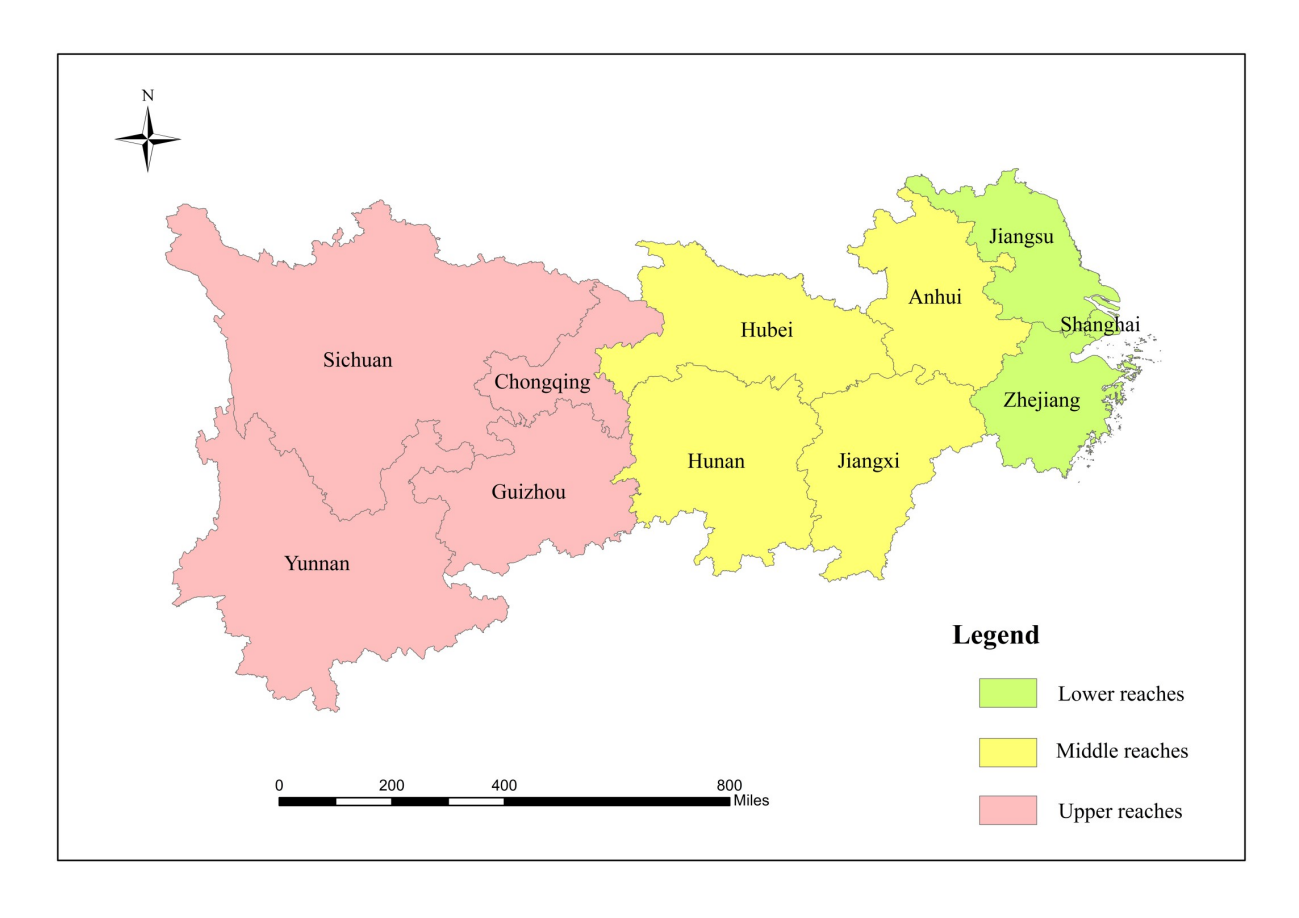
Map of the Yangtze River economic belt.

Yangtze River economic belt refers to the economic circle around the Yangtze River, covering an area of about 205.7104 km^2^, accounting for 21.27% of total land area in China. There are 11 provinces/cities including Shanghai, Jiangsu, Zhejiang, Anhui, Jiangxi, Hubei, Hunan, Chongqing, Sichuan, Yunnan and Guizhou (Fig 1). The Yangtze River economic belt is divided into upper (Chongqing, Sichuan, Guizhou and Yunnan), middle (Anhui, Jiangxi, Hubei and Hunan) and lower (Jiangsu, Zhejiang and Shanghai) reaches.

This figure is modified from CIA China map (public domain): https://www.cia.gov/library/publications/the-world-factbook/index.html. The figure only includes cities/provinces in Yangtze River economic belt, China. It’s not identical to the original image and is therefore for illustrative purpose only.

As there is no information regarding construction land area in Shanghai, this paper only considers construction land expansion in the remaining 10 provinces/cities except Shanghai [33]. Similar case can also be found from Zhang et al. [43], where Shanghai was excluded from analysis on coordination between urban construction land scale and urban population due to the same reason. For the remaining 10 provinces/cities, the research has attempted to cover a greater time period and therefore the total dataset is not small for analysis in the paper. Further, there is also published research also using similar or smaller data sets (Zhao [4], Zhang [44] and Lv [30]). Social and economic data were obtained from China statistical yearbook, China city statistical yearbook, statistical yearbooks of provinces/cities and China regional economic database in corresponding years. A few missing data were obtained by interpolation.

### Methods

**ESI and EII.** Speed and trend of construction land expansion are measured through ESI, which means the annual average expansion rate of construction land. Its absolute value represents the speed and its symbol denotes the trend. In order to facilitate comparison and analysis of spatial differences between each spatial unit, land area of each spatial unit to expansion speed was standardized, the result is EII and it indicates the expansion range of regional construction land [40]. The formulae are as follows:

$${ESI}_{i}=\frac{{}_{A_{t}-A_{0}}}{\Delta t\times A_{0}}\times 100\%$$ (1)

$${EII}_{i}=\frac{{}_{A_{t}-A_{0}}}{\Delta t\times S}\times 100\%$$ (2)

Where A_0_ is construction land area in the initial year; A_t_ is construction land area in year t;Δt is the time span; S is total land area of the region.

#### Selection of driving forces

Construction land expansion is influenced by a variety of natural, economic, social and technological factors. In short term, it is mainly impacted by government policies, technical and economic factors, while natural biology may also be a restrictive factor in long term [45, 46]. You and Yang found that economic, social and demographic factors’ impacts would increase over time, while natural factors would gradually reduce their influence on urban expansion [47]. Shao et al. [48] consider that influence of natural environmental factors on construction land expansion is relatively stable and small in short time. Therefore, this research does not include natural factors in analysis.

Specially, this research selects indicators to evaluate impacts of economic and social factors on construction land expansion. Economic factors are closely linked with urban development and population, especially in research on construction land expansion. Wen summarized the driving mechanism of construction land expansion and believed that the main driving factors can be classified into three categories: economic factors (GDP, output value of secondary and tertiary industries, income and investment level), social factors (population, urbanization) and policy factors (development policies, land planning and national guidelines), among which socio-economic factors are the main research direction [49]. Wang et al. analyzed the spatial and temporal evolution of urban construction land in the Yangtze River economic zone, and results showed that expansion of urban construction land was affected by population, GDP, industrial structure, economic activities, infrastructure and other factors [50]. Xu and Huang [39] analyzed the driving mechanism of urban expansion from the perspectives of population, economic, residents' living standard and transportation development level. Chen et al. [51] proposed that urban expansion was affected by road traffic conditions and measured urban economic development, road traffic conditions and urban population’s impacts on urban land expansion. Therefore, this research includes GDP, population at the end of the year, population urbanization rate and per capita road area to evaluate impacts of economic factors.

While influences of social factors have been explicitly acknowledged, their impacts can be more difficult to quantify. Peng and Zou [38] suggested that social policies have great impacts on social investments and thus selected social fixed asset investments to represent the policy strength when analyzing driving forces of construction land expansion. Bao & Wang [23] suggested that impacts of government policies will be reflected in socio-economic outcomes, and thus government policies can be analyzed along with other factors. Combine with the above discussions, our model also included total fixed asset investment to analyze the influence of policy factors.

Additionally, Zhang et al. [5] pointed out that while most research focuses on factors such as economy, urbanization and fixed asset investments, there is inadequate consideration of policy regulations, industrial structure and technology. More specifically for the Yangtze River economic belt, this research argues that technical and industrial structural factors can be quite influential. In 2017, about two fifth of the country’s colleges and college students are in the Yangtze River economic belt (China statistical yearbook, 2018). As there are abundant education resources along the Yangtze River economic belt and considering the national industrial development strategy implemented in the Yangtze River economic belt as mentioned previously, technical factors and industrial structure may have great influences on construction land expansion.

To sum up, eight social and economic factors are included in analysis, which are: GDP (represents urban economic development), social fixed asset investments (represents social policy strength), population at the end of the year (represents population), population urbanization rate (represents urbanization), per capita road area (represents urban road traffic conditions), number of scientific and technological professionals (represents the technical level), secondary and tertiary industry values (represents industrial structure). Construction land area was selected as the dependent variable, and its correlation with the selected driving forces was analyzed. GDP and social fixed asset investments are adjusted to actual variables according to Eq 3. Consumer price index in year 2000 was 100:

Actualvariables=Variableannualprice×100÷CPI (3)

Indexes used in this research and their data source are shown in Table 1 below:

**Table 1:** Indexes used in this research and their data source.

Index	Data sources
Construction land area	China statistical yearbook, China city statistical yearbook
GDP	China statistical yearbook, China city statistical yearbook
Social fixed asset investments	Statistical yearbooks of provinces/cities and China regional economic database
Population at the end of the year	Statistical yearbooks of provinces/cities and China regional economic database
Population urbanization rate	Statistical yearbooks of provinces/cities and China regional economic database
Per capita road area	Statistical yearbooks of provinces/cities and China regional economic database
Number of scientific and technological professionals	Statistical yearbooks of provinces/cities and China regional economic database
Secondary and tertiary industry values	Statistical yearbooks of provinces/cities and China regional economic database

#### STIRPAT model

In 1970s, ecologists and environmental scientists proposed a IPAT model to analyze relationships between economic growth and environment, that is, I = PAT [52, 53]. Subsequently, York et al. [54] added the impacts of population, affluence and technology and proposed the STIRPAT model. The model is an effective method to analyze impacts of human factors on environment pressure. STIRPAT model has attracted attention from many scholars and has been widely applied in research areas including ecological footprints, carbon emission, energy use and arable land area. The expression is as follows:

$$I=aP^{b}A^{c}{T_{3}}^{d}e$$ (4)

Where I represents environment pressure; P is population; A denotes wealth; T_3_ is technical level; a is the coefficient of the model; b, c and d are indexes of population, affluence and technology respectively; e is the residual value of the model.

According to the above discussion, this paper constructs a STIRPAT model to quantitatively measure the relationship between construction land expansion and its driving forces. This method has been widely used in other fields, such as research on influencing factors of carbon emission [55, 56], but it is seldom applied to discussions of driving force of construction land expansion. Additionally, research generally uses this model in analysis of a single city/province, while limited research using this model on regional cities where regional flow of factors can be critical. Further, applying this model to a region facilitates comparative research on construction land expansion. The model is built as follows: firstly, linear relationship between comprehensive variable and independent variable is constructed by using PCA; secondly, OLS regression is applied to evaluate the linear relationship between dependent variable and comprehensive variable; finally, result from the first step is put into that from the second step to obtain relationships between dependent variable and independent variable. The expression is as follows:

$$Y=\beta G^{a_{1}}E^{a_{2}}P^{a_{3}}R^{a_{4}}L^{a_{5}}U^{a_{6}}S^{a_{7}}T^{a_{8}}\varepsilon$$ (5)

Take the logarithm of both sides:

$$\begin{array}{l} LnY=Ln\beta +a_{1}LnG+a_{2}LnE+a_{3}LnP+a_{4}LnR+\\ a_{5}LnL+a_{6}LnU+a_{7}LnS+a_{8}LnT+Ln\varepsilon \end{array}$$ (6)

Where Y is construction land area; β is a constant; G is GDP; E is social fixed asset investments; P is population at the end of the year; R is per capita road area; L is number of scientific and technological professionals; U is population urbanization rate; S is secondary industry value; T is tertiary industry value; and ε is the random term of the model. a_1_、a_2_、a_3_、a_4_、a_5_、a_6_、a_7_、a_8_ are elasticity coefficients, representing the change of a_1_%、a_2_%、a_3_%、a_4_%、a_5_%、a_6_%、a_7_%、a_8_% of Y respectively when G, E, P, R, L, U, S, T change by 1%.

## Result and analysis

### Time series characteristics

**Fig 2:**
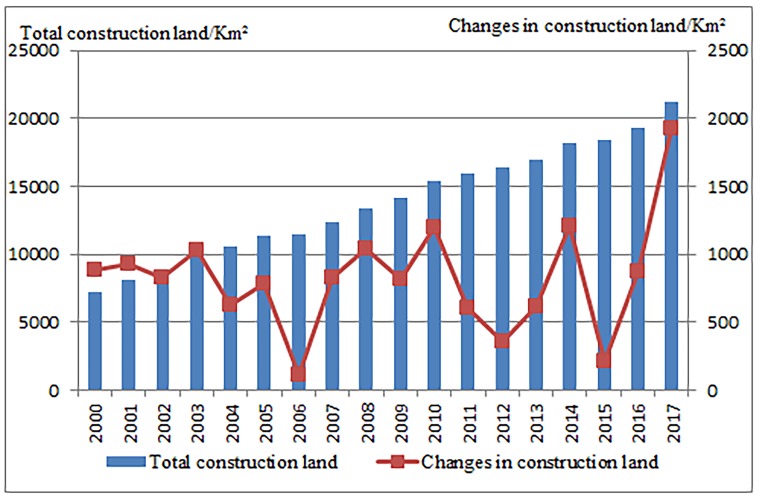
Changes in construction land area of the Yangtze River economic belt, China. Source: Chinese city statistical yearbook (corresponding years).

Changes in construction land area in provinces/cities in the Yangtze River economic belt from 2000 to 2017 are show in Fig 2 below:

With continuous economic and social development, trend of construction land expansion is obvious during this period. Overall, total construction land area has almost doubled in size, with an average annual growth rate of 7.03%. From 2000 to 2006, construction land expanded slowly, from 7184.33 km^2^ to 11500.69 km^2^, increases by 719.39 km^2^ annually. ESI and EII showed a downward trend, and changes in construction land area decreased yearly. During 2006 and 2012, construction land area increased from 11500.69 km^2^ to 16338.94 km^2^. The expansion speed increased first and slowed down, with an annual increase of 806.37 km^2^. ESI and EII showed an inverted "W" shape. From 2008 to 2009, due to impacts of the economic crisis in China, the regional market contracted seriously which limited urban development and construction land expansion. The expansion speed and intensity of construction land expansion were lower compared to adjacent years. From 2012 to 2017, construction land expansion started to increase from 16338.94 km^2^ to 21183.14 km^2^, with an average annual increase of 968.84 km^2^. ESI and EII show an upward trend, but were accompanied by large fluctuations.

Specifically regarding each provinces/cities, construction land area of Chongqing, Sichuan, Guizhou and Yunnan in the upper reaches has increased by 1099.63 km^2^, 1838.42 km^2^, 714.90 km^2^ and 865.57 km^2^ respectively, increased by 3.40, 1.85, 2.63 and 3.13 times respectively as compared to year 2000. Construction land area of Anhui, Jiangxi, Hubei and Hunan in the middle reaches increased by 1288.30 km^2^, 1019.36 km^2^, 1035.33 km^2^ and 898.13 km^2^ respectively, which are 1.72, 2.34, 0.79 and 1.11 times respectively of those of year 2000. Construction land area of Jiangsu and Zhejiang in the lower reaches increased by 3,089.54 km^2^ and 2,149.63 km^2^, 2.31 and 3.16 times respectively the size of year 2000. ESI and EII of provinces/cities in the Yangtze River economic belt show a fluctuating trend. From 2000 to 2006, ESI of construction land declined gradually, while EII remained relatively stable. ESI and EII of construction land from 2006 to 2012 are consistent with the overall trend of the Yangtze River economic belt, both of which show obvious fluctuations. As a result of the 2008 economic crisis, ESI and EII of construction land in all provinces/cities in this region declined, although differently. From 2012 to 2017, both ESI and EII showed a trend of increase—decrease—increase. Particularly, ESI of Zhejiang, Chongqing, Yunnan, Hubei and Hunan fluctuates greatly, while ESI of other provinces remain relatively stable. In terms of EII, Zhejiang, Jiangsu, Hubei, Chongqing and Hunan have greater fluctuations than other provinces.

Time series characteristics of construction land expansion are analyzed at the regional and provincial level. The results show that construction land expansion of the upper, middle and lower reaches in the Yangtze River economic belt have obvious stage characteristics, aligning with the general pattern of construction land expansion. Overall, construction land expansion in the Yangtze River economic belt can be viewed from different stages, which are rapid expansion in the early stage (2000–2006), moderate expansion in the middle stage (2006–2012) and rapid expansion in the later stage (2012–2017).

### Spatial characteristics

**Fig 3:**
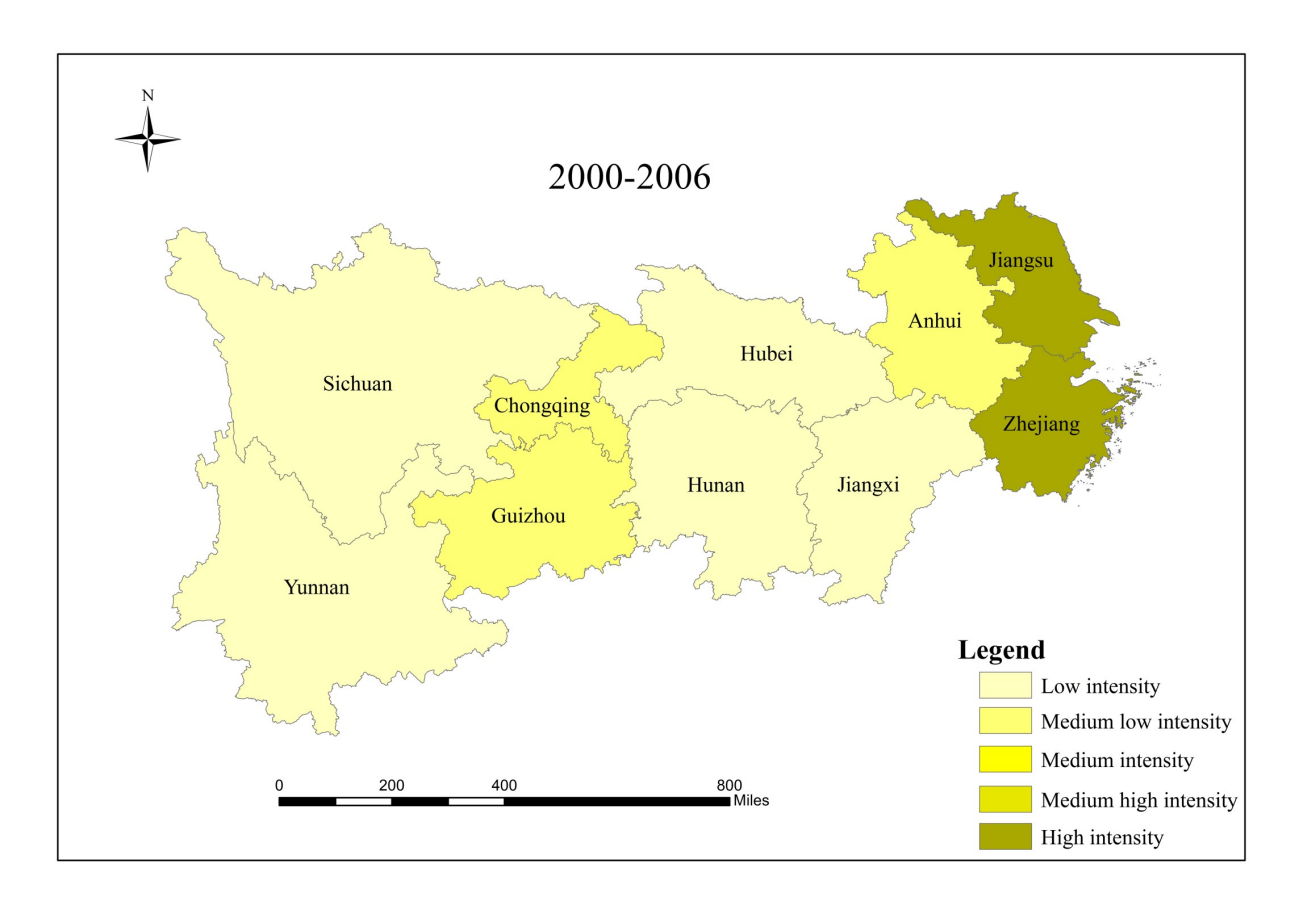
EII of construction land in provinces/cities in the Yangtze River economic belt from 2000 to 2006.

**Fig 4:**
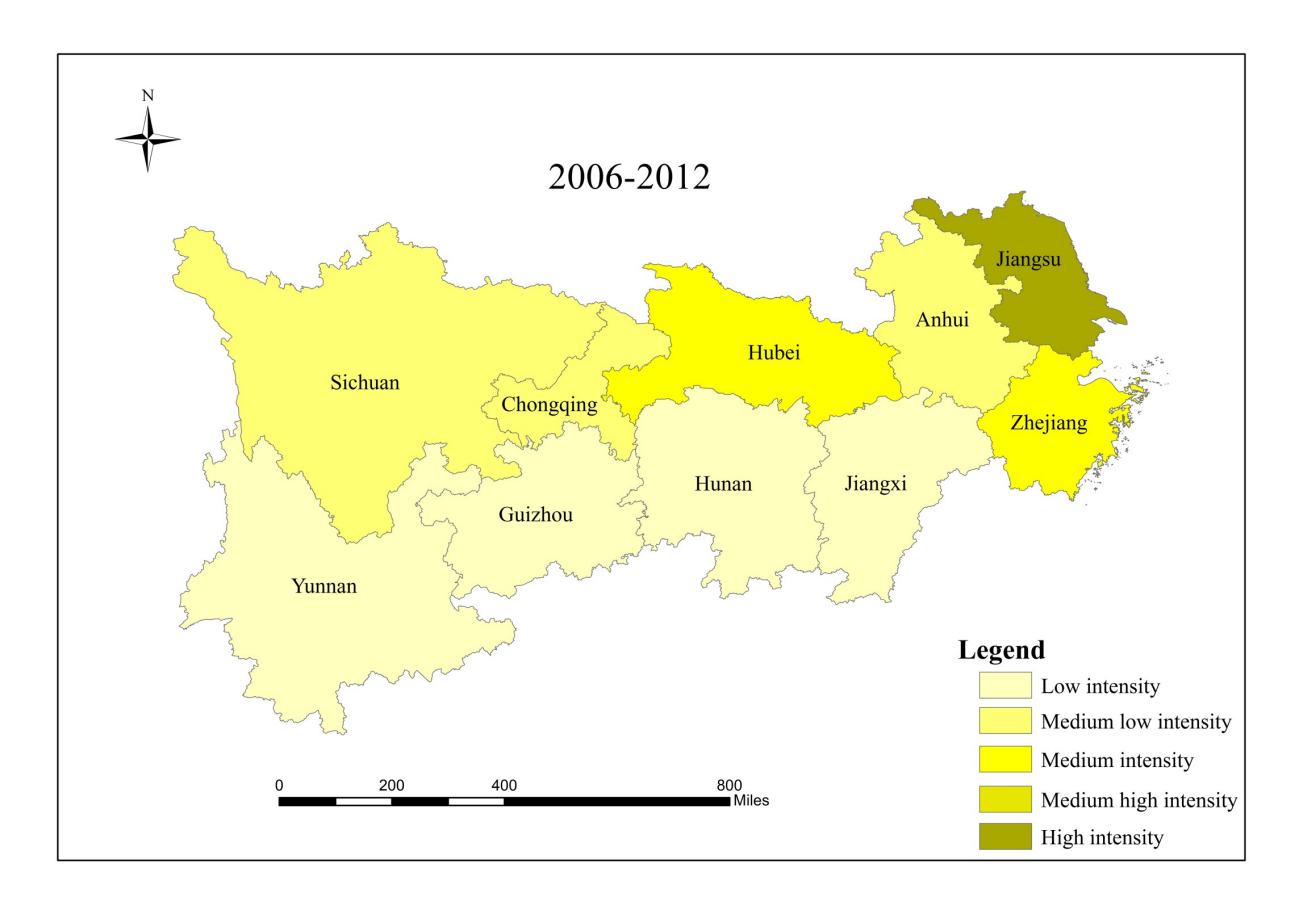
EII of construction land in provinces/cities in the Yangtze River economic belt from 2006 to 2012.

**Fig 5:**
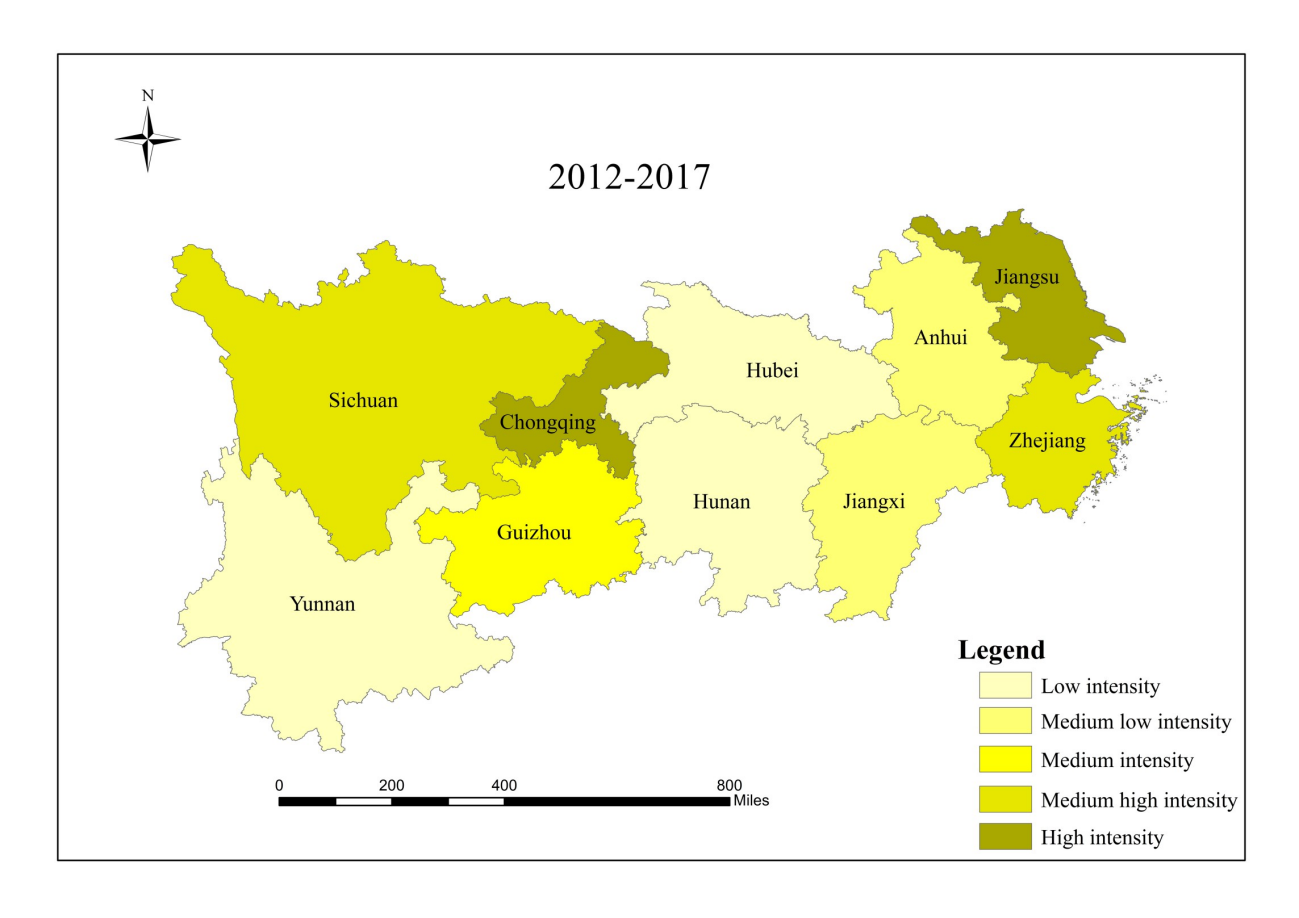
EII of construction land in provinces/cities in the Yangtze River economic belt from 2012 to 2017.

**Fig 6:**
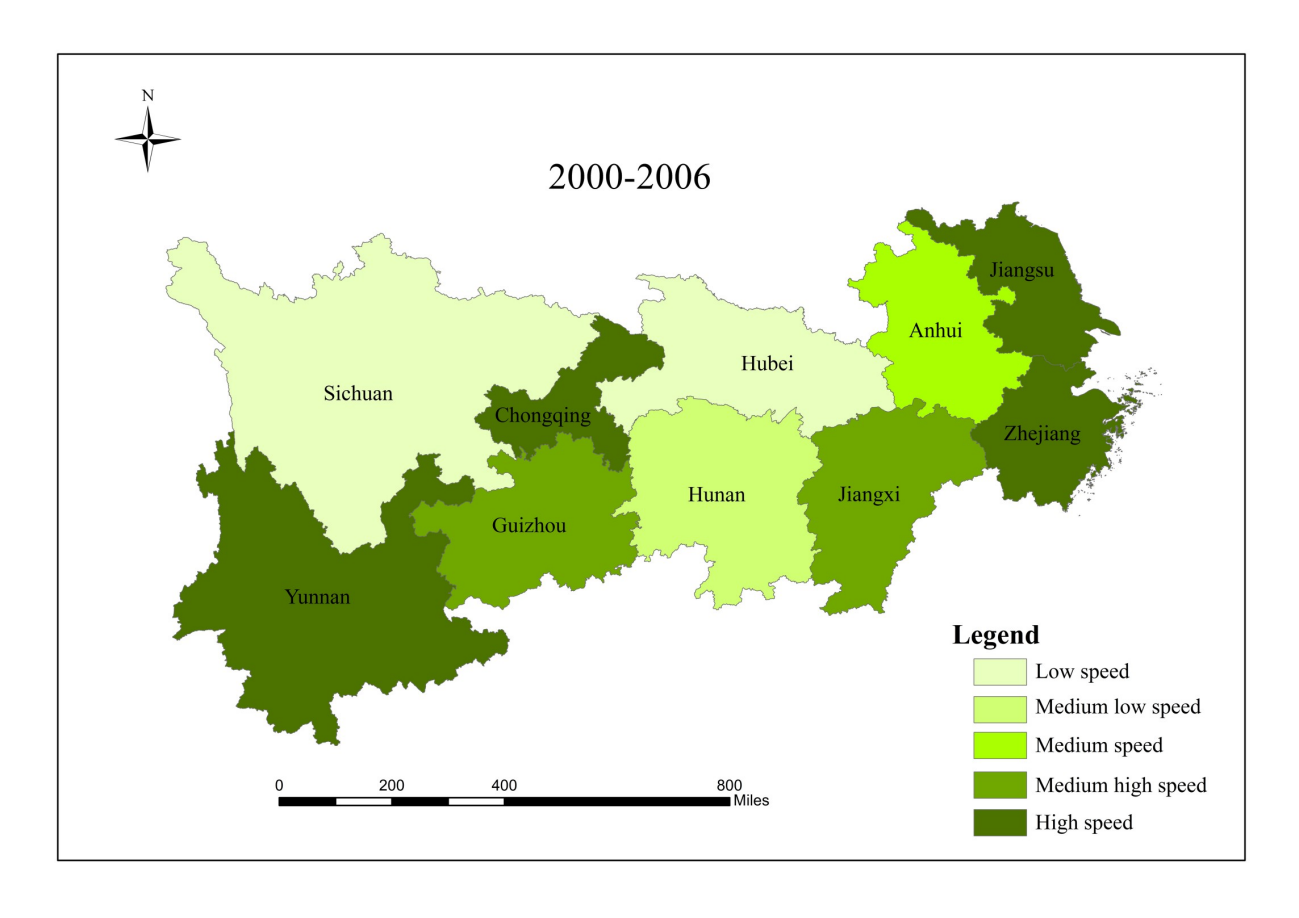
ESI of construction land in provinces/cities in the Yangtze River economic belt from 2000 to 2006.

**Fig 7:**
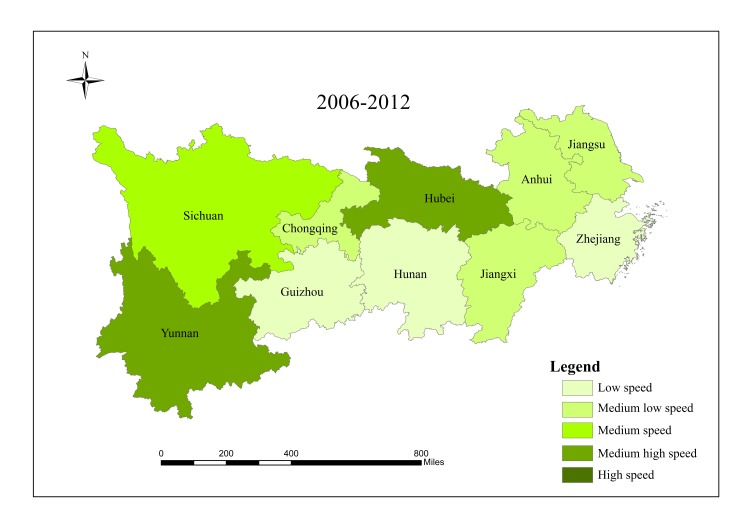
ESI of construction land in provinces/cities in the Yangtze River economic belt from 2006 to 2012.

**Fig 8:**
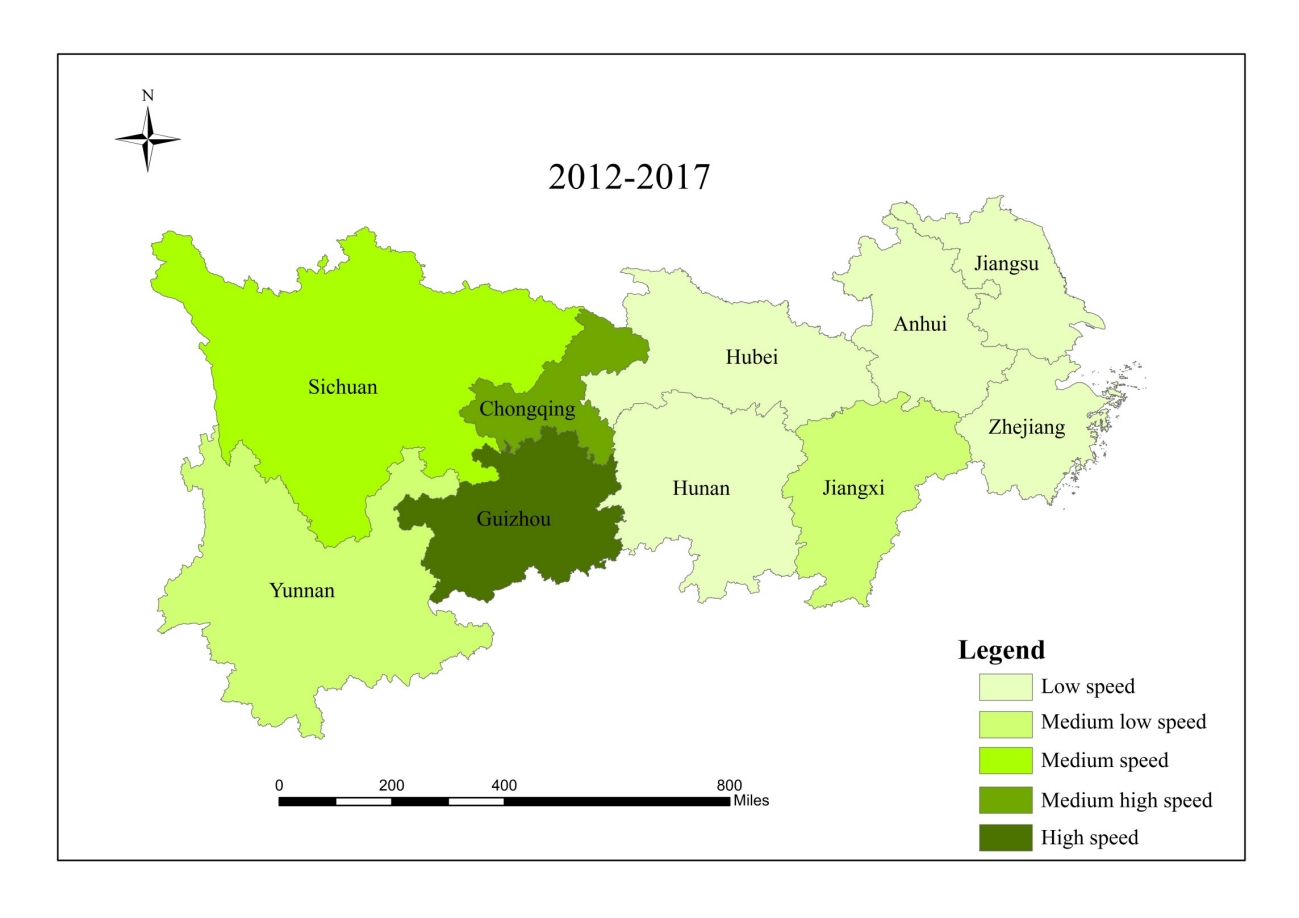
ESI of construction land in provinces/cities in the Yangtze River economic belt from 2012 to 2017. Source: Figs 3–8 are all based on Fig 1 and obtained by using Arcgis mapping software according to the analysis results.

When analyzing spatial characteristics of construction land expansion, the research period is divided into three intervals: 2000–2006, 2006–2012 and 2012–2017. According to Andrea [57], ESI and EII of construction land in 10 provinces/cities were classified into five grades (Table 2), and spatial characteristics of construction land expansion were analyzed in Figs 3–8.

**Table 2:** Classification standard of construction land expansion.

ESI	EII	The classification standard
High speed	High intensity	(average + Standard value,+∞)
Medium high speed	Medium high intensity	(average +0.5*Standard value, average + Standard value]
Medium speed	Medium intensity	(average, average +0.5*Standard value]
Medium low speed	Medium low intensity	(average–0.5*Standard value, average]
Low speed	Low intensity	(–∞,average–0.5*Standard value]

The results show that there are significant regional differences in ESI and EII of construction land in the Yangtze River economic belt. Construction land expands along with the social and economic development. Overall, construction land in the lower reaches expands faster than that in the middle and upper reaches, and shows a trend of transferring from high-speed-high-intensity expansion area to middle and upper reaches, showing obvious discrepancies in spatial patterns.

From 2000 to 2006, the lower reaches demonstrate characteristics of high-speed and high-intensity expansion of construction land, and ESI and EII of construction land were both higher than the regional average. Hubei in the middle reaches and Sichuan in the upper reaches are characterized by low-speed and low-strength expansion. The upper reaches of Guizhou, Yunnan and Chongqing have a higher expansion rate, while the expansion intensity lags behind other provinces and cities simultaneously. Expansion speed and intensity are moderate in the middle reaches.

From 2006 to 2012, spatial differences among all provinces/cities regarding construction land expansion intensity and speed started to decline. Except Jiangsu which still maintained high intensity expansion, and Hubei which developed from low intensity in the first stage to medium intensity expansion in the second stage, expansion intensity of other provinces/cities decreased compared with those in the first stage. For ESI, Sichuan and Hubei developed from low expansion in the first stage to medium expansion in this period, while expansion speed of other provinces/cities decreased. Additionally, there was a significant change in the lower reaches of Zhejiang, from a high-speed-high-intensity expansion to a low-speed-low- intensity expansion.

From 2012 to 2017, except Anhui, Jiangxi, Hubei, Hunan and Yunnan, construction land in other provinces/cities expanded significantly and gradually shifted to the upper reaches. During this period, the economy of Guizhou, Sichuan and Chongqing developed rapidly, which resulted in changing mode of land use and promoting upgrading of industrial structure and played active role in rapid expansion of construction land in the upper reaches. Overall, construction land in the research region shows spatial characteristics of first expansion of the lower reaches, rapid expansion of the upper reaches in the middle and late period, and stable expansion of the middle reaches. Moreover, differences in spatial pattern within this region declined gradually, and construction land expansion tends to be stable and more balanced.

### Driving forces analysis

#### Analysis of partial correlation

Correlation analysis was used to analyze the correlation between the selected eight driving forces and construction land area. Then significance tests are carried out to determine whether the selected factors can be used as the driving forces. The results show that correlation coefficients of the eight driving forces and construction land area are all above 0.6, and all have passed the bilateral significance test at the level of 0.01. Therefore, the selected factors can be included in the model for analyzing driving forces of construction land expansion.

#### PCA

Firstly, logarithm of the original data of dependent variables and explanatory variables is obtained. Secondly, in order to ensure data comparability, logarithmic data were input into SPSS22.0 software for standardized processing to eliminate dimensional differences among explanatory variables. The processed data were represented as ZY, ZG, ZE, ZP, ZR, ZL, ZU, ZS and ZT, respectively. Finally, standardized data were input into SPSS22.0 software for PCA. Partial correlation between variables was examined by KMO tests. In general, PCA can be performed if KMO statistics are greater than 0.7. Correlation analysis shows that KMO test values of all provinces/cities are greater than 0.7 (Table 3), and the significance of spherical test statistics is less than 0.01, indicating that factor analysis is appropriate. Through PCA, two principal components, namely comprehensive variables, are obtained, which are respectively represented by F1 and F2. Results show that degree of interpretation of the two comprehensive variables to original variables are higher than 90% (Table 3), indicating that fitting degree between comprehensive variables and original variables is relatively good. Correlation coefficients between comprehensive variables and each original variable obtained from PCA can be seen from Table 4.

**Table 3:** Degree of interpretation of original variables by comprehensive variables and KMO test value.

	Jiangsu	Zhejiang	Anhui	Jiangxi	Hubei	Hunan	Chongqing	Sichuan	Guizhou	Yunnan
Degree of interpretation(%)	99.064	98.387	96.416	99.028	97.354	91.900	97.398	90.734	97.869	99.311
KMO test value	.813	.812	.771	.881	.815	.807	.814	.796	.818	.737

**Table 4:** Correlation coefficients between comprehensive variables and original variables.

		ZG	ZP	ZE	ZU	ZL	ZR	ZS	ZT
Jiangsu	F1	.147	.143	.141	.147	.099	.141	.144	.158
	F2	.017	-.011	-.028	.024	1.002	-.006	-.010	.110
Zhejiang	F1	.201	.168	.215	.162	.123	-.459	.194	.183
	F2	-.112	-.041	-.143	-.026	.048	1.219	-.098	-.072
Anhui	F1	.150	.099	.147	.156	.133	.147	.142	.162
	F2	.007	.998	-.007	.056	-.011	-.004	-.055	.108
Jiangxi	F1	-.170	.664	1.225	1.173	.739	-1.748	.755	-1.926
	F2	.365	-.521	-1.117	-1.063	-.603	2.041	-.618	2.230
Hubei	F1	.113	.480	.114	.171	-.416	-.739	.053	.161
	F2	.072	-.464	.071	-.012	.369	1.250	.157	.004
Hunan	F1	.164	.240	.156	.172	-.107	.229	.160	.137
	F2	-.015	-1.018	.014	-.049	-.013	-.248	.001	.072
Chongqing	F1	.212	-.753	.431	.404	-.488	.584	.292	.066
	F2	-.046	1.181	-.307	-.276	.773	-.501	-.142	.127
Sichuan	F1	.172	-.106	.172	.169	.039	.151	.176	.163
	F2	-.017	.698	-.023	.003	.488	-.128	-.060	.043
Guizhou	F1	.098	.638	.167	.195	.440	.375	.106	.023
	F2	.096	-1.141	-.007	-.049	-.420	-.330	.084	.205
Yunnan	F1	.241	.463	.265	.366	.081	-1.072	.163	.226
	F2	-.118	-.436	-.153	-.296	.106	1.726	-.007	-.097

#### OLS regression

OLS is used for regression analysis between ZY (explained variable) and F1 and F2 (explanatory variables). The results show that, the fitting model R^2^ of ZY, F1 and F2 in provinces/cities are all greater than 0.9, and significance of t tests is lower than 0.01, indicating that the model fitted well (Table 5).

**Table 5:** Regression coefficient of dependent variable and comprehensive variables and R^2^.

	ZY									
	Jiangsu	Zhejiang	Anhui	Jiangxi	Hubei	Hunan	Chongqing	Sichuan	Guizhou	Yunnan
F1	.979	.936	.975	.754	.779	.960	.829	.941	.905	.828
F2	-.175	.173	-.022	.642	.558	.096	.540	.244	.346	.501
R^2^	.989	.906	.951	.982	.919	.931	.974	.944	.939	.936

According to Tables 4 and 5 and formulae (6), elasticity coefficient of driving forces can be obtained (Table 6). Elasticity coefficients are significantly different as economic and social development levels and resource endowments differ greatly within this region. Driving forces of each province/city are graded according to level of the elastic coefficient, and the biggest three driving forces are selected to conduct statistical analysis. The results show that social fixed asset investments and population urbanization rate, GDP as well as secondary industry value are important driving forces in six, five as well as four provinces/cities, respectively. Other forces have just a few common impacts. Therefore, social policy strength, urbanization, urban economic development, and industrial structure are major forces driving construction land expansion in this region.

**Table 6:** Elasticity coefficient of driving forces in construction land expansion.

	G	E	P	R	L	U	S	T
Jiangsu	0.1409	0.1418	0.1430	0.1401	-0.0785	0.1389	0.1422	0.1354
Zhejiang	0.1689	0.1503	0.1764	0.1467	0.1232	-0.2187	0.1647	0.1591
Anhui	0.1461	0.0751	0.1437	0.1504	0.1297	0.1431	0.1393	0.1558
Jiangxi	0.1066	0.1658	0.2059	0.2022	0.1699	-0.0076	0.1725	-0.0203
Hubei	0.1283	0.1147	0.1283	0.1262	-0.1176	0.1225	0.1286	0.1275
Hunan	0.1560	0.1327	0.1507	0.1607	-0.1041	0.1966	0.1534	0.1385
Chongqing	0.1511	0.0132	0.1917	0.1863	0.0135	0.2138	0.1656	0.1233
Sichuan	0.1573	0.0703	0.1561	0.1593	0.1559	0.1104	0.1511	0.1633
Guizhou	0.1217	0.1823	0.1485	0.1594	0.2530	0.2253	0.1247	0.0920
Yunnan	0.1402	0.1649	0.1427	0.1543	0.1200	-0.0226	0.1311	0.1385

Population urbanization rate is the main driving force for construction land expansion in Chongqing, Sichuan, Guizhou and Yunnan, indicating that urbanization has a great influence in upstream provinces/cities. The main reason is that economic and social development of the upper reaches lags behind that of the middle and lower reaches on the whole, and construction land expansion of the former is mainly promoted by urbanization process. By comparison, construction land expansion in Anhui, Jiangxi, Hubei and Hunan are further affected by GDP. These provinces are in central China, which are geographically superior to provinces/cities in the upper reaches. Therefore, these provinces may also be influenced by radiation effects of various resources in surrounding areas and thus economic and social development have significant positive impacts on construction land expansion. The common driving forces of Jiangsu and Zhejiang in the lower reaches are different from those in the middle and upper reaches. Their main driving forces are social fixed asset investments and secondary industry value. Jiangsu and Zhejiang are located in eastern China. Different from middle and upper reaches, urbanization plays a weak role in driving construction land expansion. The main reason is that for these two provinces, economic development and urbanization are relatively high and thus have limited impacts on construction land expansion.

In addition to common driving forces, each province/city also have different important driving forces. Construction land expansion in Jiangsu and Yunnan is further greatly affected by population. Population of these two provinces grows faster and this thus creates further demands for residential and infrastructure land, promoting construction land expansion. Tertiary industry value is the most influential factor in Anhui and Sichuan, followed by urbanization and GDP. This can be explained from the fact that Anhui and Sichuan are undergoing industrial structure upgrading and developing their tertiary industry. In this case original land area is inadequate to meet new demands, resulting in construction land expansion. Road traffic conditions also impact greatly on construction land expansion in Hunan and Chongqing. For this two provinces, road traffic conditions have improved significantly in recent years, with construction of subways, high-speed rails and urban roads, thus leading to the construction land expansion. However, for Guizhou, there is no common driving forces with other provinces/cities, and instead, technical level, road traffic conditions and population are major forces. Guizhou’s growth in number of technical personnel is only smaller than that of Zhejiang. This means that improvement in technical level is also likely to bring population inflow and promote industrial development, which partly explains the continuous increase of construction land area.

## Conclusions and discussions

In this paper, spatiotemporal characteristics of construction land expansion in Yangtze River economic belt from 2000 to 2017 were studied through ESI and EII. Then, based on a STIRPAT model, driving forces were quantitatively measured with PCA and OLS regression. The results show that: (1) there is a clear expansion pattern regarding the time sequence in provinces/cities of the Yangtze River economic belt, with rapid expansion in the initial stage, moderate expansion in the middle stage and rapid expansion in the later stage. (2) Spatial analysis shows first expansion in the lower reaches in early stage, rapid expansion of the upper reaches in the middle and later stage, and steady expansion of the middle reaches throughout the research period. Moreover, differences in spatial pattern between provinces and cities narrow gradually, and construction land expansion tends to be increasingly stable and more balanced. (3) There are statistical significant correlations between construction land expansion and GDP, social fixed asset investments, population at the end of the year, population urbanization rate, per capita road area, number of scientific and technological professionals as well as secondary and tertiary industry values. Of these factors, GDP, social fixed asset investments, population urbanization rate and second industry value are important common driving forces of provinces/cities of the Yangtze River economic belt. For each provinces/cities, different economic development levels and resource conditions have resulted in differences in important driving forces. In addition to common driving forces, population, urban road traffic conditions and tertiary industry value are also important driving forces in certain provinces/cities.

The research results show that construction land expansion in the Yangtze River economic belt has obvious temporal and periodical characteristics during the research period. This conclusion is consistent with Zhang & Zhu [32], which indicates that provinces and cities of the Yangtze River economic belt do show obvious characteristics of stages and regional differences in construction land expansion. In terms of driving forces of construction land expansion, results from this paper show that regional GDP, social fixed asset investment, urbanization level and secondary industry value are important common driving forces of provinces and cities in the Yangtze River economic belt, indicating that these factors play a major role in promoting the expansion of construction land. This is also in line with many other research (Peng & Zou [38], Liu et al [46], He et al. [28]) focusing on individual cities. The research results of the Yangtze River delta urban agglomeration in this paper are similar to those of other individual cities by relevant scholars, which indicated that this method is suitable for the study of urban agglomeration in the Yangtze River economic belt. This further indicates that the influence of inter-regional factor flow on the expansion of construction land is similar to that of the study on a single city without considering factor flow. This means that similar conclusion can be obtained when the models of STIRPT and combine PCA with OLS are used to urban agglomeration in the Yangtze River economic belt.

However, this research displays a slightly different picture regarding expansion patterns, as compared with existing literature. Results of Li et al.[57] show that expansion of construction land in the Yangtze River economic belt in the past 30 years presents a linear increasing process, which is mainly manifested as "deceleration (1986–1996)—rapid (1996–2006)—deceleration (2006–2016)". In comparison, this paper shows an expansion trend of rapid expansion in the early period (2000–2006), deceleration expansion in the middle period (2006–2012) and rapid expansion in the late period (2012–2017). This discrepancy may be explained by selection of research area and indexes. Li et al. [57] only focuses on representative cities in the Yangtze River economic belt, while this paper considers the whole Yangtze River economic belt. In addition, industrial structure factors that were seldom considered by scholars were added to this study. With increased mobility of inter-regional factor and strengthened industrial policies, transformation and upgrading of industrial structure will play a significant role in promoting construction land expansion, as shown by Zhao [4]. This also explains why this study shows rapid construction land expansion from 2012 to 2017.

By applying the models of STIRPT and combine PCA with OLS to urban agglomeration in the Yangtze River economic belt, this research arrives at similar conclusions with existing research mostly focuses on single cities. This adds to existing literatures that inter-regional factor flow follows impacts of factors that have significant influences on construction land expansion. It thus has significant policy implications as Chinese cities are putting growing emphasis on coordinated development of urban agglomerations.

Further, by including indexes of industrial structure, this research unveils the significance of secondary industry values, this is particularly important as industry upgrading is a major focus of China’s economic development and the Yangtze River economic belt is acting as a pioneer in this regard. This further means that conclusions from this research can be important references to other areas of China where urban construction land expansion issue is concerned and in the meantime industry upgrading is undergoing.

However, as data for Shanghai is missing, research findings on the Yangtze River economic belt might not be fully comprehensive. Further, this research does not include natural factors’ influences on construction land expansion as argued previously in this paper that their impacts are not significant in short period. If longer time span is concerned and to enable better applicability of the research results, more work is needed to find ways to deal with these issues.

## Supporting information

S1 Data Data tables of provinces and cities along the Yangtze river economic belt (2000–2017). (XLSX)

S1 Source of statistics (DOCX)
