# Supplementary material for: Spatiotemporal characteristics and driving forces of construction land expansion in Yangtze River economic belt, China
Source: PLoS One. 2020 Jan 24;15(1):e0227299. doi: 10.1371/journal.pone.0227299 (PMC6980553; doi:10.1371/journal.pone.0227299)
Supplement: S1 Source of statistics — (DOCX) [file pone.0227299.s002.docx]

**The source of statistics**

Cnki data statistics website：<http://data.cnki.net/Yearbook/Navi?type=type&code=A>

EPS data platform website：<http://olap.epsnet.com.cn/>

Jiangsu statistics website：<http://tj.jiangsu.gov.cn/col/col4009/index.html>

Zhejiang statistics website：<http://tjj.zj.gov.cn/col/col1525563/index.html>

Anhui statistics website：<http://tjj.ah.gov.cn/tjjweb/web/tjnj_view.jsp?_index=1>

Jiangxi statistics website: <http://tjj.jiangxi.gov.cn/id_tjnj201803120104397238/column.shtml>

Hubei statistics website：<http://data.hb.stats.cn/CityData.aspx?DataType=67&ReportType=3>

Hunan statistics website：<http://tjj.hunan.gov.cn/tjsj/tjnj/>

Chongqing statistics website：<http://data.tjj.cq.gov.cn/publish.htm?code=A01>

Sichuan statistics website：<http://tjj.sc.gov.cn/tjcbw/tjnj/>

Guizhou statistics website：<http://stjj.guizhou.gov.cn/tjsj_35719/sjcx_35720/gztjnj_40112/tjnj2018/>

Yunnan statistics website：<http://www.stats.yn.gov.cn/tjsj/tjnj/>
